# What is moving where? Infants’ visual attention to dynamic objects may assist with processing of spatial relations

**DOI:** 10.3389/fpsyg.2023.1261201

**Published:** 2024-01-18

**Authors:** Jihye Choi, Youjeong Park

**Affiliations:** Department of Child Development and Family Studies, Seoul National University, Seoul, Republic of Korea

**Keywords:** infants, spatial categorization, support relations, dynamic objects, eye-tracking

## Abstract

**Introduction:**

A central question in infant spatial cognition concerns *how* infants form abstract categories of spatial relations such as support (*on*) and containment (*in*). Prior work suggests two different possibilities regarding the role of attention to objects in infants’ formation of abstract categories of spatial relations: Attention to objects may compete with (and thus hamper) attention to the spatial relations between them, or assist with encoding of the spatial relation information. Using eye-tracking, we examined how infants’ visual attention to objects related to their successful formation of an abstract category of support relations (i.e., an object *on* another).

**Methods:**

Thirty-eight 8-month-old infants’ eye movements were recorded during a support categorization task, where infants were habituated to four dynamic events depicting support relations (e.g., resting a block on a box) and then presented with test events that depicted either a support or containment relation with objects that they had seen or not seen in the habituation phase. Based on their looking time to the familiar versus novel spatial relation in the test, infants were classified into two groups: categorizers, who formed an abstract category of a support relation, and non-categorizers, who did not do so.

**Results:**

During their initial phase of learning (i.e., the first habituation trial), categorizers paid greater attention to the object moved by a hand (i.e., the dynamic object) in comparison to non-categorizers, whereas their attention to the static object or their gaze shifts between the two objects did not differ. In addition, when presented with novel objects in a novel spatial relation after habituation, only categorizers displayed asymmetric attention between the objects, attending to the dynamic object more than the static object. Gaze shifts and attention to the concave area (i.e., hole) of the container did not differ between categorizers and non-categorizers.

**Discussion:**

These findings suggest that infants’ focused attention to an object in motion may play a key role in young infants’ spatial category learning, and support the idea that attention to objects can assist with encoding of the spatial relational information.

## 1 Introduction

Surrounded by numerous objects and the spatial relations they compose, infants begin to form categories of spatial relations before knowing their labels (e.g., Behl-Chadha and Eimas, 1995; Casasola et al., 2003; Quinn, 2007; Casasola and Park, 2013). Around 6 to 7 months, they are able to form abstract categorical representations of containment relations (Casasola et al., 2003) and the relations of *above* versus *below* (Quinn et al., 1996), as evidenced by generalization of the spatial relations to objects that are novel to them (Casasola and Cohen, 2002). Further, infants of 8 months show evidence for the ability to categorize support relations (i.e., an object *on* another) although they do so in highly limited circumstances (Park and Casasola, 2015; Park and Choi, 2023).

The early emergence of spatial categorization raises the question of *how* infants (learn to) categorize spatial relations. The cognitive mechanism underlying infant spatial categorization has been a central topic in infant spatial cognition research (e.g., Casasola, 2005b; Casasola and Park, 2013; Park and Casasola, 2015). An information processing approach, which views infants as taking in information from the environment and processing the information through attention, perception, encoding, memory, etc., has served as an apt theoretical framework for such research (Casasola, 2011; Oakes et al., 2011). For instance, the approach has revealed that infants earlier in development attend to the simpler, objects’ featural information in discriminating visual events, whereas they later attend to the spatial relations to discriminate the events (Quinn et al., 1996; Casasola and Cohen, 2002). Also, the regression-under-stress principle of the approach (Cohen and Cashon, 2006) successfully explains the finding that infants capable of processing spatial relations in visual input revert to processing the input at the simpler, featural level when the visual input contains an increased number of objects (Casasola, 2005b; Cohen and Cashon, 2006).

A potential key process in infants’ spatial categorization from an information processing view is to go beyond encoding objects and encode a spatial relation between the objects in a scene. There is evidence suggesting that paying attention to spatial relations requires additional cognitive processes compared to the requirements for recognizing object features such as color (Logan, 1994; Holcombe et al., 2011). Yet, little is known about what the additional processes are or what facilitates attention to a spatial relation between objects.

One factor that might be linked to infants’ attention to spatial relation information is infants’ attention to objects. Prior research suggests two different possibilities with respect to how infants’ attention to objects relates to their encoding of a spatial relation. First, attention to objects may compete with attention to relations. Given that the attentional resource for information processing is limited (e.g., Vecera and Farah, 1994), objects’ grabbing more of the infant’s attention might leave less attention available for spatial relations, thus hampering infants’ encoding of spatial relations. Consistent with this possibility, 14-month-old infants who viewed support events depicted by diverse pairs of objects, which presumably drew much attention to the objects *per se*, were less able to discriminate between the familiar spatial relation (support) and a novel spatial relation (containment) than their peers who viewed support events described by only two pairs of objects (e.g., Casasola, 2005b).

Alternatively, attention to objects may benefit encoding of a spatial relation. Experiments with 8- and 10-month-old infants have shown that settings that would stimulate infants’ interest in objects are advantageous for forming a support category. Specifically, infants of 7.5 to 8.5 months successfully categorized support relations when they were habituated to dynamic support events depicted with richly decorated blocks, but not when they were habituated to the same support relations depicted with monotonous, plain blocks (Park and Casasola, 2015). Also, 10-month-old infants, unlike 14-month-olds, formed a category of support relations when they viewed more diverse pairs of objects during habituation, but not when they viewed only two pairs of objects (Casasola and Park, 2013). These findings suggest that infants’ attention to objects may not necessarily harm their ability to encode spatial relations, at least for those of younger ages. Aligned with this possibility, Casasola (2011) has proposed that a bottom-up cognitive process may underlie infants’ categorization of spatial relations: infants may attend to a spatial relation only after processing and becoming sufficiently familiarized with the objects in the spatial relation. If it is the case, then infants’ attention to objects may be a prerequisite for attending to the spatial relation that they constitute, serving as a gateway to processing spatial relational information.

Another unresolved question is whether infants’ attention to different objects that constitute a spatial relation would equally relate to their spatial categorization. Typical tests of infant spatial categorization present infants with dynamic events involving pairs of objects, with each pair consisting of a dynamic object (i.e., an object carried by a hand to a new location) and a static object (i.e., an object fixed on the ground; e.g., Casasola and Cohen, 2002; Park and Choi, 2023). Ample evidence indicates that dynamicity attracts infant attention; infants preferably attend to a dynamic stimulus over a static one (e.g., Volkmann and Dobson, 1976; Slater, 1989). Also, from their first hours after birth, infants are sensitive to visual cues indicating whether a motion is self-propelled or not, and use this information for object individuation and agency attribution (Di Giorgio et al., 2017; Decarli et al., 2020). Therefore, although both objects are relevant to a spatial relation, a dynamic object likely draws more attention from infants than the still object and might have a larger effect on infants’ spatial categorization whether it is a facilitating effect or a hampering effect.

Furthermore, research findings suggest that infants’ observation of a moving object might provide them with opportunities to attend to spatial information. Specifically, when a human hand or a geometric shape moves on an agentive trajectory, infants around 7 months spatially orient their attention in congruence with the object’s movement direction (Wronski and Daum, 2014). Also, viewing a moving object, six- to seven-month-olds represent its path, defined as the trajectory of an entity relative to a reference point (e.g., over, under, and past; Pulverman et al., 2013), and accurately predict the spatiotemporal dynamics of the object when the object is temporarily invisible (Gredebäck and von Hofsten, 2004). Thus, it is possible that attention to a dynamic object aids infants’ encoding of destination (i.e., a final position of the object) as well, consequently assisting with the encoding of its position relative to another object. Indeed, in multiple object tracking tasks, adults encoded the final position of an object more precisely when they were instructed to attend to motion of the objects (Howard et al., 2017). Moreover, infants as young as 5 months demonstrate the ability to comprehend the goal object of an approaching motion (Woodward, 1998; Luo and Baillargeon, 2005), which may serve as a base for understanding the goal position of a carried object. By the age of 12 months, infants preferably attend to the destination of a path to its origin (Lakusta et al., 2007), suggesting the salience of destination in infants’ motion perception. Thus, the findings together point to the possibility that changes in object location over time invite infants to the encoding of spatial relation between objects. Viewing a stationary object might be less effective in promoting the encoding of spatial relation than viewing a dynamic object, due to the lack of change in its location over time. If so, then infants’ proficiency in selectively attending to a moving object may be closely linked to successful processing of spatial relation in dynamic events.

Also, research has suggested that attentional *shifts* between objects may play an important role in adults’ processing of spatial relations in a visual scene (Holcombe et al., 2011; Franconeri et al., 2012; Yuan et al., 2016). In an EEG study by Franconeri et al. (2012), adults were requested to judge the left/right relationship between shapes while trying to attend to both shapes simultaneously; their ERP patterns indicated sequential attentional shifts to each shape. In contrast, adults were able to identify object colors with no attentional shift toward the objects signaled by EEG (Luck and Ford, 1998). Furthermore, an eye-tracking study reported that adults’ voluntary gaze shifts between objects accelerated their judgment of the objects’ relative position, but not their judgment of the object color (Yuan et al., 2016). Therefore, it has been claimed that spatial relationships among objects in a novel scene can be represented when computation of relative positions occurs through shifts of attentional selection between an object and a reference point (Franconeri et al., 2012; Yuan et al., 2016). In light of the link between attentional shift and processing of spatial relations in adults, visual attention shifts between the relevant objects might help infants successfully encode spatial relations, although the association has been evidenced only for static scenes thus far.

Lastly, previous research evaluated infants’ categorization of spatial relations based on their looking times to a habituated relation event versus a novel relation event (e.g., Casasola and Cohen, 2002; Casasola, 2005a,b; Park and Casasola, 2015). If infants in the test phase look longer at the novel relation event than the habituated relation event when the objects in both events are novel, it has been interpreted as evidence for the formation of an abstract categorical representation of the habituated relations. The rationale is that in an infant-controlled habituation paradigm, infants view the habituation stimuli until the stimuli are sufficiently familiar for them at their own visual processing pace (Colombo et al., 1991), and increase their looking time in response to novelty, not familiarity (Hunter and Ames, 1988; Cohen, 2004). Thus, infants’ longer looking in a containment event than a new support event following habituation to support events would be a sign that they find the containment relation more novel than the new support relation. However, to ensure this interpretation, a possibility should be ruled out that the longer looking time to the containment event merely reflects infants’ notice of (attention to) the concave area of the container (i.e., the hole), which is a perceptual feature distinct from the flat surface of the static objects in support events. Otherwise, the infants’ longer looking at the novel relation event could be reduced to more interest in a new perceptual feature of the stationary object (container). Thus, infant visual attention during a novel relation event in the test phase need to be examined, focusing on infants’ attention to the concave area.

### 1.1 The current study

The main goal of this study was to examine how infants’ attention to objects relates to the successful abstract categorization of support relations. Specifically, we aimed to test whether (1) more attention to a moving object, (2) more attention to a static object, and (3) more sequential shifts of attention between a moving object and a static object would relate to successful categorization of support relations in 8-month-old infants. In addition, we aimed to examine whether infant attention to the concave area when presented with a containment event in the test phase would relate to their successful categorization of support relations.

To achieve the goals, we utilized eye-tracking and an infant-controlled habituation paradigm to record infants’ eye movements during a typical test of infant support categorization. It would allow us to classify the infants into categorizers, who formed an abstract category of a support relation, and non-categorizers, who did not do so, and compare their visual attention patterns. As described earlier, although prior studies suggested the link between infant attention to objects and encoding of spatial relations (Vecera and Farah, 1994; Casasola, 2005b,^2011^; Casasola and Park, 2013; Park and Casasola, 2015), they had a limitation that they did not directly measure infants’ attention to objects. Rather it was assumed that infants would pay greater attention toward objects when there was a higher level of variability or a greater degree of perceptual richness of objects. Thus, an eye-tracking study that measures infants’ fixations onto the objects during a spatial categorization task would allow us to more directly examine infants’ attention to objects and its relationship with infants’ successful formation of an abstract spatial category.

The age of infants and the category of relations were selected on the basis of previous findings that 8-month-old infants categorized support relations when the objects were perceptually rich but regressed when they were perceptually sparse (Park and Casasola, 2015). Possibly, infants of this age are not proficient at selectively attending to a support relation yet; for these infants, visual attention targeted on the relevant objects might serve as a gateway to attending to a support relation. Thus, our hypotheses were as follows. First, infants who successfully categorize support relations (categorizers) would pay more of their attention to the relevant objects (the dynamic and the static objects) than those who fail to categorize the relations (non-categorizers), with a larger difference in attention to the dynamic object. Particularly, categorizers may show a more noticeable preference for the dynamic object over the static object in processing a dynamic spatial event than non-categorizers. Second, categorizers who succeed at a support categorization task would exhibit more eye gaze shifts between the dynamic and static objects during a spatial categorization task than non-categorizers. Third, categorizers would not fixate the concave area more than non-categorizers when they are presented with a containment event in the test.

We selected two time points to capture infants’ attention to objects: the first trial of the habituation phase and the test trial presenting novel objects in a novel relation. The first habituation trial was chosen to represent an early stage of learning, considering the importance of the initial stage of processing a scene for guiding the subsequent search in the scene (Võ and Henderson, 2010). Additionally, infants were expected to be highly engaged in visual exploration of the scene when they first encountered it, providing an ideal opportunity for comparison between categorizers and non-categorizers. Next, we selected the test trial presenting novel objects in a novel relation because the trial would allow us to determine whether categorizers and non-categorizers would differ in their attention to the concave area of the container. It would also enable us to examine how infants allocated attention to the objects during the process of noticing the novel spatial relation when both the objects and the relation were novel. A test trial presenting familiar objects in a novel relation would be less ideal for testing infants’ attention to the objects because the infants would have already become familiarized with the objects through the habituation process, and pay little attention to the objects.

## 2 Materials and methods

### 2.1 Participants

Forty-three 8-month-old infants were recruited from the Seoul metropolitan area, South Korea. Five of them were excluded due to crying. Thus, data from 38 infants (22 males, *M* age = 8.0 months, range = 7.5–8.6 months) were submitted to the final analysis. All participants were typically developing full-term infants with normal vision and hearing. Most parents had a college degree or higher.

To determine the sample size, previous studies that utilized similar habituation paradigms to assess infants’ spatial categorization (Casasola and Park, 2013; Park and Casasola, 2015; 16 infants per group) and those that integrated habituation with eye-tracking measures (Johnson et al., 2004; Gaither et al., 2012; 7 to 21 infants per group) were used as reference. Also, when designing the study, we conducted an *a priori* power analysis using G*Power 3.1.9.4 (Faul et al., 2009). It revealed that 23 infants per group were needed to detect a group difference of effect size 1.09 with α = 0.05 and power = 0.90. The effect size was obtained from Johnson et al. (2004), where a group difference was found in visual attention between high-performing and low-performing preverbal infants during an object perception task. However, our achieved sample size fell short of the planned because data collection was interrupted by safety measures taken in response to the COVID-19 pandemic. A *post hoc* power analysis using the observed effect size estimate (ranging from 0.70 to 0.83), the achieved sample size (*n* = 18, and *n* = 20 for two groups), and alpha level of 0.05 revealed a range of statistical power being from 0.6 to 0.7, which is lower than the field benchmark of 0.8.

### 2.2 Stimuli

**FIGURE 1:**
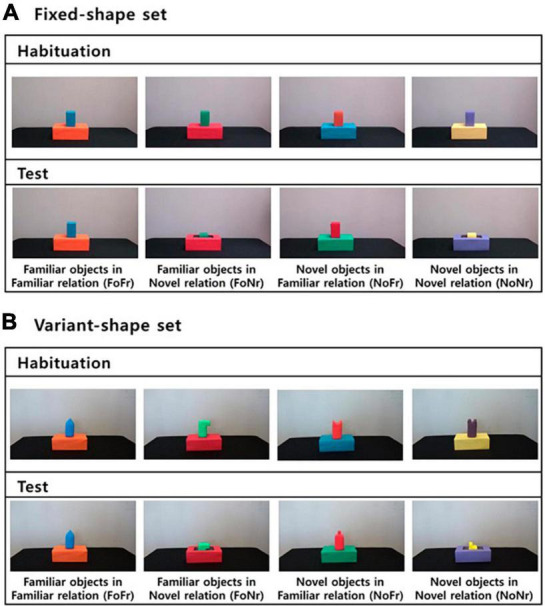
Snapshots of sample video stimuli in the support categorization task. **(A)** Fixed-shape set, **(B)** Variant-shape set.

A set of dynamic events depicting a support relation or a containment relation were filmed with a digital video camera and edited into 7-s videos. Each video began with a block and a larger box set side by side on a table. After 1 s, a hand entered the scene, grasped the block, and placed it either on or in the larger box. The hand exited the scene immediately, and the last 2 s of the video showed the final relation between the two objects. The blocks were monochrome rectangular blocks approximately 6 cm x 6 cm x 10 cm in their dimensions, with the top being either flat or of variant forms. The larger boxes were solid-color (blue, green, orange, purple, red or yellow) rectangular boxes that were 23.5 cm × 10.5 cm × 8.5 cm in their dimensions. Twenty-five infants viewed the flat-top blocks and 13 infants viewed the blocks of the variant top shapes (see Figure 1). The effect of the block type was not significant and thus was not considered in the present study.

### 2.3 Apparatus

A Tobii Pro X3-120 Eye Tracker was used for data collection. The eye tracker was attached to a 22-inch Dell monitor that presented video stimuli. It has an average gaze position error of 0.5° and a spatial resolution of 0.2°. Eye movements were recorded binocularly at a sampling rate of 120 Hz. A table covered by black cloth supported the monitor at the infants’ eye level (approximately 55 cm from the floor). A Logitech HD webcam was placed below the monitor to allow the experimenter to observe the infants’ looking behavior. The webcam was concealed from the infants by black cloth with a small hole for the camera lens.

### 2.4 Procedure

Infants were tested either in a laboratory on campus or at their home. When infants were tested in non-laboratory settings, the environment was kept as similar as possible to the lab settings. More specifically, the experimenter brought a set of equipment including a portable table covered with black cloth, a chair, a screen monitor, and a camera to the testing places. An independent, quiet room was selected to prevent any interruption by others or any external noises. In order to minimize visual distractions, the monitor was positioned in front of a blank wall, and potentially attention-grabbing items such as artworks and toys nearby were removed prior to the test. Infants’ guardians provided written informed consent about their child’s participation and completed a demographic survey. Then infants were seated on the guardian’s lap approximately 65 cm from the monitor. Guardians were requested to wear opaque glasses throughout the testing session to avoid noise to the eye-tracking data by the guardians’ gazes. They were also instructed not to talk to the infants or point to the screen until the end of the testing. An experimenter controlled the eye-tracker and observed the infants’ looking behaviors either in an adjoining room at the laboratory or under the table covered with black cloth at home visit. Infants’ behaviors during the testing session were video-recorded. The Habit 2 (version 2.2.7) program (Oakes et al., 2019) was used to present the visual stimuli and record infant looking times.

The support categorization task took approximately 5 min per infant including calibration. Calibration was conducted at the beginning of the testing session using a 5-point fixation procedure in Tobii Pro Lab software and repeated if necessary. Infants were then presented with a support categorization task, which consisted of three phases: pretest, habituation, and test. The pretest was provided to familiarize infants with the test settings. During the pretest, infants viewed a stuffed animal moved by a hand in a zigzag path until they lost interest and looked away or they reached the maximum looking duration of 30 s. A valid look was initiated by 1 sec of continuous looking at the stimulus, and was terminated by 1sec of continuous looking away from the stimulus (Colombo and Horowitz, 1985). On the screen, a green circle that expanded and contracted with a chiming sound was used as the attention-getter, appearing at the center of the screen prior to every trial. As soon as the infants looked at the screen, the experimenter ceased the attention getter and started the trial. Every trial lasted up to 30 s if not stopped by infants’ looking away for one continuous sec or more. The pretest was followed by a habituation phase, during which participants repeatedly viewed four different events depicting a support relation. Presentation order of the four habituation events was designated using Latin square technique. Each infant’s average looking times were automatically calculated per window of three consecutive trials, as in the prior research on infant support categorization (e.g., Casasola and Park, 2013; Park and Casasola, 2015). The habituation phase ended once infants’ average looking time for the window dropped more than 50% from that of the first window, following a convention of infant-controlled habituation procedure (e.g., Cohen, 1976, 2004; Casasola and Ahn, 2018). A maximum of 18 trials were presented in the habituation phase, adjusted from the maximum of 20 trials in previous research that tested 8-month-olds’ support categorization (Park and Casasola, 2015) to slightly shorten the habituation phase while maintaining the number of windows (6 windows). The adjustment was based on our observation in pilot that infants tended to get fussy and quit participation before entering the test phase when they had to watch the maximum number of trials. Given that it took an average of 10.19 trials for 8-month-old infants in Park and Casasola (2015) to habituate to support events (*SD* = 4.56), we considered a maximum of 18 trials reasonable.

Then, the test phase began and presented infants with four test trials in a fixed order. Adopting a procedure used in multiple infant habituation studies (Casasola and Cohen, 2002; Casasola et al., 2003, 2009), the first test trial displayed an event seen during habituation, therefore referred to as the familiar-objects-familiar-relation test trial (FoFr). The next three trials showed the events that were novel with respect to the spatial relation, objects, or both: familiar-objects-novel-relation (FoNr), novel-objects-familiar-relation (NoFr), and novel-objects-novel-relation (NoNr) test trials. For the purpose of the present study, infant looking times to the NoFr and NoNr trials were critical because if infants formed an abstract categorical representation of the support relations, they would look longer at the novel spatial relation than the familiar relation in the test phase even when the objects were novel. Note that we did not randomly assign the infants into two groups. As we grouped the infants into categorizers and non-categorizers based on their looking times in the test trials with novel objects, we aimed to keep the test order consistent across the two groups by using the uniform sequence of test trials for all infants. For each infant, a novel relation preference score was calculated by dividing their looking time to the novel relation trial (NoNr) by the sum of looking times to the familiar relation trial and the novel relation trial (NoFr + NoNr). Infants whose novel relation preference score was greater than 0.5 were classified as categorizers, while the others were considered non-categorizers. All study protocols were approved by the Institutional Review Board of Seoul National University.

### 2.5 Data processing

#### 2.5.1 Fixation analysis

**FIGURE 2:**
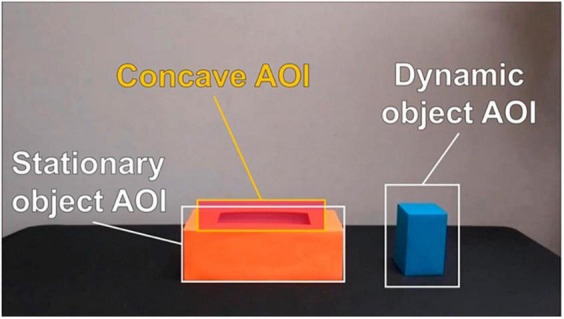
Areas of interest (AOIs). Note that the concave AOI was used in analyses of the novel relation (containment) test trial only.

Fixation was defined as stable looking where the velocity of directional shifts of the eye was under 30° per second. To measure infants’ visual attention on the objects, fixations on three areas of interest (AOIs) were analyzed: the dynamic object, the stationary object, and the concave area of the stationary object (see Figure 2). The dynamic object AOI was manually determined and checked for each participant to ensure that they precisely captured the fixations on the dynamic object. Also, for the NoNr trial, the size of the dynamic object AOI was adjusted to the visible part of the dynamic object when the object was inserted into the stationary object, being between 250 × 350 px and 250 × 150 px. The size of the stationary object AOI was fixed at 550 × 250 px. The concave AOI was a rectangular shape covering the hole of the stationary object with a size of 240 × 120 px, and it was analyzed only for the novel relation (containment) trial with a novel object pair (the NoNr trial). Tobii Pro Lab software calculated the numbers and durations of infants’ fixations in each AOI. The longer duration of fixations in AOI is interpreted as deeper and more effortful processing of information or more interest in the area (Just and Carpenter, 1980; Holmqvist et al., 2011). The number of fixations in AOI, which is found to be greater in the informative areas (Holmqvist et al., 2011), was also analyzed to reaffirm the results on the duration of fixations. The fixation measures were converted to proportions as a function of fixations on the whole screen. Independent samples t-tests were used to compare the fixation measures between categorizers and non-categorizers. Any trials that had no fixations were discarded. Specifically, for four infants, fixation data were recorded for the first habituation trial but not for the test trial; these infants were excluded from the fixation analyses for the NoNr test trial.

#### 2.5.2 Gaze switch analysis

The gaze switch between the dynamic object and the stationary object was operationalized as a fixation on the stationary object AOI following a fixation on the dynamic object AOI, or vice versa. To examine infants’ gaze switches between the dynamic and the stationary objects, we exported each infant’s sequence of fixation, saccade, and AOI hit (i.e., whether or not a look was on each AOI) from Tobii Pro Lab software. We then counted instances of gaze switches between the objects in each trial for each participant. Although we planned to analyze the mean number of gaze switches, the gaze switch between the dynamic object and the stationary object turned out to be scarce. Therefore, we used the proportion of infants who had at least one switch between the objects for comparison of categorizers and non-categorizers.

## 3 Results

Among our participants, 18 infants were grouped into categorizers, whose average novel relation preference score being 0.67 (*SD* = 0.13), and 20 infants were found to be non-categorizers (*M* novel relation preference score = 0.42, *SD* = 0.07). Preliminary analyses confirmed that infants’ looking time to the first habituation trial did not differ between categorizers (*M* = 22.32s, *SD* = 7.87s) and non-categorizers (*M* = 17.47s, *SD* = 9.35s), *t* (36) = 1.72, *p* = n.s. The number of fixations on the screen per second, a measure of active processing of information on the screen (Johnson et al., 2004), was also comparable between the categorizers (*M* = 2.11, *SD* = 0.77) and the non-categorizers (*M* = 2.11, *SD* = 0.73), *t* < 1, *p* = n.s. Thus, the two groups did not differ in the extent to which they paid attention to the stimulus event at the beginning of the habituation phase.

### 3.1 Eye movements during the first habituation trial

#### 3.1.1 Fixations on the dynamic versus stationary objects during the first habituation trial

Our goal was to examine whether categorizers and non-categorizers would differ in their allocation of visual attention to a dynamic object and a stationary object in early processing of the support events. Since the total numbers and durations of fixation on the screen varied across individuals, we used the proportionate number of fixations and the proportionate duration of fixations.

**FIGURE 3:**
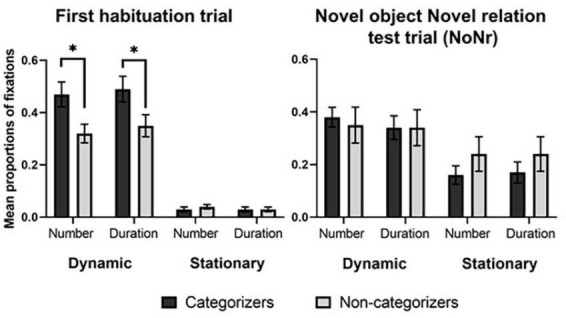
The mean proportions of fixation number and duration on the dynamic and stationary objects in the first habituation trial and the novel relation test trial with novel objects (NoNr) by group. Error bars indicate ±1 standard error of the means, *p < 0.05.

**FIGURE 4:**
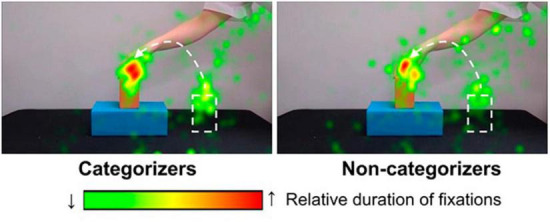
Heat maps during the first habituation trial by group.

First, we conducted group comparisons on infants’ number of fixations and duration of fixations in the dynamic object during the first habituation trial. As shown in Figure 3, the proportionate number of fixations on the dynamic object was significantly greater in the categorizers (*M* = 0.47, *SD* = 0.20) than in the non-categorizers (*M* = 0.32, *SD* = 0.16), *t* (36) = 2.65, *p* = 0.01. Likewise, the proportionate duration of fixations in the dynamic object was higher in the categorizers (*M* = 0.49, *SD* = 0.21) than in the non-categorizers (*M* = 0.35, *SD* = 0.19), *t* (36) = 2.05, *p* = 0.048. Thus, infants who later succeeded in forming an abstract categorical representation of support relations allocated proportionately more attention to the dynamic object in early processing of the habituation events, compared to those who later failed in support categorization. More concentrated fixations on the dynamic object in categorizers than non-categorizers are also shown in the heat maps (Figure 4). The heat map for categorizers contained a larger red spot on the top portion of the moving object (e.g., the orange block in Figure 4) and displayed more concentration of colored spots than the heat map for non-categorizers, indicating categorizers’ more focused interest in viewing the moving object in this early phase of learning.

Next, infants’ visual attention to a stationary object was examined. Both categorizers and non-categorizers allocated a lower proportion of attention to the static object relative to the dynamic object, based on the proportionate duration of fixations, paired *t* (17) = 8.93, *p* < 0.001 for categorizers, and paired *t* (19) = 7.77, *p* < 0.001 for non-categorizers. Neither the proportionate number (*M*_*cat*_ = 0.03, *SD* = 0.04, *M*_*non–cat*_ = 0.04, *SD* = 0.04) nor the proportionate duration (*M*_*cat*_ = 0.03, *SD* = 0.04, *M*_*non–cat*_ = 0.03, *SD* = 0.04) of fixations on the stationary object differed between the two groups, *t*s < 1, *p*s = n.s. That is, categorizers who later succeeded and non-categorizers who later failed to categorize support relations equally attended to the static object in the initial phase of habituation.

#### 3.1.2 Gaze switches between the dynamic and stationary objects during the first habituation trial

Although we planned to analyze the mean number of gaze switches among categorizers and non-categorizers, instances of gaze switch between the dynamic and the stationary objects were rarely observed. Therefore, we analyzed the proportion of infants who showed at least one gaze switch between the two objects. Seven out of 18 categorizers (38.9%) and 6 out of 20 non-categorizers (30.0%) switched their gaze between the dynamic and the static objects at least once. The difference was not significant, χ^2^ = 0.33, *p* = n.s., thus failing to provide evidence that infants who later successfully formed an abstract categorical representation of support relations were more likely to show gaze switches between the relevant objects than those who later failed to do so.

### 3.2 Eye movements during the test trial with novel objects depicting a novel relation (NoNr test trial)

#### 3.2.1 Fixations on the dynamic versus stationary objects during the NoNr test trial

Group comparisons were implemented to test the patterns of visual attention in infants who have succeeded or failed at forming an abstract categorical representation of support relations when encountering novel objects depicting a novel relation in the test phase. Two infants in the categorizer group and two infants in the non-categorizer group were excluded from these analyses due to a lack of fixation data for this test trial, caused by the infants’ fussy movements. Thus, fixation data from 16 infants in the categorizer group and 18 infants in the non-categorizer group were analyzed.

First, we examined whether categorizers allocated more attention to each of the relevant objects than non-categorizers. No significant group difference was found in the proportionate number (*M*_*cat*_ = 0.38, *SD* = 0.15, *M*_*non–cat*_ = 0.35, *SD* = 0.29) and the proportionate duration (*M*_ cat_ = 0.34, *SD* = 0.18, *M*_*non–cat*_ = 0.34, *SD* = 0.29) of fixations on the dynamic object, *t*s < 1, *p*s = n.s. Thus, when a novel relation was presented with a novel pair of objects after habituation, the extent to which infants attended to the dynamic object did not differ by group. Next, we compared infants’ visual attention to a stationary object. No significant difference was found in the proportionate number of fixations on the stationary object between categorizers and non-categorizers (*M*_*cat*_ = 0.16, *SD* = 0.14, *M*_*non–cat*_ = 0.24, *SD* = 0.28), *t* < 1, *p* = n.s. The proportionate duration of fixations also did not differ between the groups (*M*_*cat*_ = 0.17, *SD* = 0.16, *M*_*non–cat*_ = 0.24, *SD* = 0.28), *t* < 1, *p* = n.s. Thus, when presented with a novel relation event in the test phase, the extent to which infants attended to the stationary object did not differ by groups, either. Additional analyses of the remaining test trials also revealed no group differences in infants’ proportional fixations on either object (see Supplementary Material).

Given that the group comparison for attention to each object yielded no significant difference in fixations, we further evaluated infants’ relative allocation of attention to the dynamic versus static object. Categorizers allocated a lower proportion of attention to the static object relative to the dynamic object, based on the proportionate duration of fixations, paired *t* (15) = 2.71, *p* = 0.02. For non-categorizers, however, there was no significant difference between attention to the static object and the dynamic object, *t* (17) = 1.05, *p* = n.s. Thus, only categorizers displayed differential allocation of attention between the two objects, attending to the object in motion more than the static object. Categorizers’ tendency to fixate more on the dynamic object than the static object was consistently found in all the other test trials (see Supplementary Material). In contrast, non-categorizers displayed varying patterns in the allocation of fixations: They focused more on the dynamic object than the stationary one during the familiar relation test trials, but this tendency was less evident during the familiar object novel relation test trial.

In addition, we examined whether categorizers and non-categorizers differently allocated attention to the concave area of the stationary object to determine whether the categorizers’ prolonged looking at the novel relation event could be due to their longer looking at the concave area, a new feature of the larger box. For the proportionate number of fixations, the group comparison yielded no difference (*M*_*cat*_ = 0.12, *SD* = 0.11, *M*_*non–cat*_ = 0.23, *SD* = 0.29), *t* (22) = 1.55, *p* = n.s. (degrees of freedom calculated based on Welch’s correction for unequal variances and rounded). Neither did it for the proportionate duration of fixations (*M*_*cat*_ = 0.11, *SD* = 0.11, *M*_*non–cat*_ = 0.24, *SD* = 0.28), *t* (23) = 1.70, *p* = n.s. (again, degrees of freedom calculated based on Welch’s correction and rounded). Thus, the two groups’ attention to the concave area did not differ proportionally.

#### 3.2.2 Gaze switches between the dynamic object and the stationary object during the NoNr test trial

Eight of 18 categorizers (44.4%) and 8 out of 20 non-categorizers (40.0%) showed at least one gaze switch between the dynamic object and the stationary object in the NoNr trial, χ^2^ = 0.08, *p* = n.s. Thus, we found no evidence that infants who had successfully formed an abstract categorical representation of support relations were more likely to display gaze switches between the relevant objects while exploring a novel relation in the test phase.

### 3.3 Comparison between the first habituation trial and the NoNr trial in attention to the dynamic object and the stationary object

Categorizers’ proportionate duration of fixations on the dynamic object significantly decreased from the first habituation trial to the novel relation test trial, *t* (15) = 2.50, *p* = 0.03. However, there was no significant decrease in the proportionate duration of fixations on the dynamic object in non-categorizers, *t* < 1, *p* = n.s. Thus, only categorizers’ attention to the dynamic object dropped from the first habituation trial to the novel relation test trial. With regard to fixations on the stationary object, infants’ proportionate duration of fixations significantly increased from the first habituation trial to the novel relation test trial for both categorizers, *t* (15) = −3.74, *p* = 0.002, and non-categorizers, *t* (17) = −3.15, *p* = 0.01.

## 4 Discussion

This study aimed to investigate the relationship between infants’ attention to objects and their successful formation of an abstract category of support relations in order to gain insights into infants’ cognitive processes to categorize spatial relations. We analyzed eye movements during the first habituation trial and a novel relation test trial of a support categorization task in 8-month-old infants who succeeded in forming an abstract category of support relations (categorizers) and those who did not (non-categorizers).

### 4.1 The relation between infants’ attention to objects and their encoding of a support relation during the learning phase

By examining infants’ fixations and gaze switches during the first habituation trial, we aimed to explore what may assist infants’ going beyond processing object features to encode spatial relations during the learning phase. Specifically, we hypothesized that infants who successfully formed a support category would be more attentive to the relevant objects, particularly the dynamic object, than those who failed to do so. Our data from the first habituation trial supported this hypothesis.

During the first habituation trial, both categorizers and non-categorizers allocated greater attention to a dynamic object (47% of fixations for categorizers and 32% for non-categorizers on average) than a static object (an average of 3% of fixations for both groups), confirming the well-established finding that infants prefer to fixate moving than stationary stimuli (Volkmann and Dobson, 1976; Slater, 1989). However, the two groups differed in the amount of attention allocated to the dynamic object: Categorizers made more frequent and longer fixations on the dynamic object than did non-categorizers. We obtained no evidence that categorizers allocated more attention to the stationary object than non-categorizers: Fixations on the stationary object were comparably short in the two groups. Thus, the two groups differed in attention to the dynamic object but not attention to the static object, suggesting asymmetry between 8-month-old infants’ attention to the dynamic object and that to the stationary object in their links to successful categorization of support relations.

Our second hypothesis was that infants who successfully categorized support relations would display more gaze shifts between the dynamic and the static objects during the learning phase. However, we found no evidence for a relationship between infant gaze switches and successful categorization of support relations. In the first habituation trial (and the test trial showing a novel relation), infants’ gaze shifts between the dynamic and the stationary objects were rarely observed: Less than half of the infants showed at least one gaze shift between the relevant objects, in both groups.

Together, the results from the analyses of the habituation trial suggest that infant attention to the dynamic object, but not attention to the static object or sequential shifts of attention between the dynamic and static objects, may assist infants in going beyond focusing on object features to encode the spatial relation between objects. Our finding regarding attention to the dynamic object is in line with previous findings indicating that an object’s movement (that is, changes in location over time) arouses infants’ attention to spatial information. While prior research demonstrates that a moving object arouses infants to attend to direction (Gredebäck and von Hofsten, 2004), path (Pulverman et al., 2013; Wronski and Daum, 2014), and destination (Woodward, 1998; Luo and Baillargeon, 2005; Lakusta et al., 2007) of a movement, the present research suggests that attending to a moving object can aid infants’ encoding of a spatial relation that the object constitutes with another object at its final position.

In light of the relevance of the static object to the spatial relation, the lack of group differences in attention to the stationary object during the first habituation trial is somewhat unexpected. It may be due to the paucity of fixations on the stationary object during this early learning phase. In dynamic events, motion dominantly draws infants’ attention (Volkmann and Dobson, 1976; Slater, 1989); the presence of an object in motion may prevent a static object from being encoded. Indeed, eight-month-old infants who were familiarized with a crossing event discriminated a new static ground from the familiarized one (e.g., a railroad and a bridge) only when the crossing event was presented as a still shot with no motion in the human figure crossing the ground (Göksun et al., 2009). Possibly, attention to a static object might play a significant role in infant support categorization if the relations are presented in a static image in the habituation phase.

Similarly, our finding regarding gaze shifts should be interpreted carefully considering the methodological differences. The finding seems contrary to the sequential shift account (Franconeri et al., 2012), which emphasizes the importance of a sequential shift of attention in the mechanism of spatial relation processing. However, previous studies that found attentional shifts between the relevant objects in the processing of spatial relation employed static images including two objects placed apart from each other (e.g., a sausage above a box in Burigo and Knoeferle, 2015; a red circle above a blue circle in Yuan et al., 2016); our study used dynamic videos of a hand carrying an object onto or into another object at a natural speed. This movement of one object may have made it difficult for a gaze shift to occur. Since the target spatial relation was not known until the moved object was rested on the stationary box, gaze switching while the moving object was approaching to the stationary object may have been ineffective in encoding the spatial relation. In addition, once the objects formed the target relation, they were in contact with each other. Due to the proximity and the overlapping portions between the objects, a gaze shift might not have been necessary for encoding the spatial relation at this moment. Future research could investigate whether categorizers would make more gaze shifts between the dynamic and the static objects if a moving object is held for some moments at a distance from a static object before contact.

### 4.2 The relation between infants’ attention to objects and their noticing of a novel spatial relation during the test phase

By examining infant fixations and gaze switches during the test trial depicting a novel relation with a novel pair of objects, we aimed to obtain insights into how infants’ attention to objects would relate to their noticing of a novel spatial relation after habituation. Additionally, we aimed to determine whether categorizers’ longer looking to the novel relation trial could be reduced to their preference for a novel object feature.

Our hypothesis that infants who have successfully formed a support category during habituation would be more attentive to the relevant objects, particularly the dynamic object, than those who have failed to do so was not supported. Unlike the first habituation trial, categorizers’ fixations on both objects were comparable to those of non-categorizers in this test trial. The lack of group difference resulted from categorizers’ reduced attention to the dynamic object compared to the first habituation trial (49% to 34% for categorizers; 35% to 34% for non-categorizers), as shown by our cross-trial comparisons. The decrease in attention to the dynamic object likely reflects a decrease in infant attention to irrelevant details of the object, which is expected to occur as infants have formed an abstract categorical representation of support relations (e.g., one thing on another). While infants who have formed a support relation category may focus their attention on the object’s relational role, non-categorizers, who have not formed the abstract categorical representation yet, may maintain their level of attention to the object’s features such as color and shape.

The lack of group difference in fixations on the static object can be understood by considering the increases in fixations on the stationary object observed in both groups in this trial, compared to the first habituation trial (3% to 17% for categorizers and 3% to 24% for non-categorizers). The increase suggests that infants noticed the new feature in the stationary objects (the presence of a hole) across groups. Importantly, the absence of group difference in fixations on the concave area of the static object indicates that categorizers’ prolonged looking to the containment event cannot be reduced to their greater interest in the concave area or better noticing the perceptual difference between containers and supporting boxes. Also, the lack of difference is unlikely attributed to paucity of fixations on the stationary object, since both categorizers and non-categorizers showed an increased attention to a stationary object compared to the habituation trial.

Despite the lack of group difference in fixations, our data reveal that the two groups differently distributed their attention between the two objects: Categorizers maintained their focused fixations on the dynamic object relative to the stationary object during this test trial, whereas non-categorizers showed no systematic bias in fixation allocation between the objects. The categorizers’ allocation of attention favoring the dynamic object over the stationary one resonates with the idea of advanced information processing skill to selectively attend to more relevant parts of the events (Slater, 1989; Amso and Johnson, 2006), which might facilitate infants’ perception of spatiotemporal information, and therefore their notice of the spatial relation between objects. Thus, again, the importance of relevant objects appear to be asymmetric.

We found no evidence for the relationship between gaze switch and successful categorization of support relations. Gaze shifts between the dynamic and the stationary objects were observed in less than half of the infants, regardless of whether they distinguished the containment relation from the support relation at the test phase. Similar to the habituation trial, object dynamicity and proximity may be the underlying reasons. However, it is noteworthy that our finding does not imply that dynamic scenes always discourage gaze shifts between objects. Infants shift their gaze between two simultaneously moving parts during an object unity task (Johnson et al., 2004). Thus, infants’ sequential gaze shifts between objects may occur more often and be more important to encoding of a spatial relation between objects that are in motion and in distance.

Altogether, our findings support the possibility that infants’ attention to objects benefits their attention to spatial relation information, with the promoting effect limited to the attention on a moving one. At the same time, our evidence provides one way to better understand previous research where 8- and 10-month-old infants were more successful at categorizing support relations under conditions that encouraged attention to objects (Casasola and Park, 2013; Park and Casasola, 2015). Those studies have left it unclear whether attention to the dynamic and stationary objects played equal roles in spatial categorization, as the level of variability or the degree of perceptual richness was manipulated through both objects. Our discovery implies that the heightened attention to the moving objects likely has aided infants in those studies in attending to spatial relations. Thus, for infants, especially young infants whose skills to selectively attend to a support relation are still developing, attending to a relevant object in motion may serve as a crucial starting point for encoding and eventually categorizing the spatial relation. This explanation is in line with the bottom-up process (Casasola, 2011) in that it underscores processing of a component object as a prerequisite for processing of relations among objects.

Our study has several limitations. First, it should be noted that our result does not demonstrate the causality between attention to objects and spatial relation processing. It is unclear whether the categorizers succeeded in forming the category of support because they paid attention to objects, or they had third advantages linked to both their attention to objects and successful spatial categorization. It is also plausible that infants more interested in spatial information are more attentive to the dynamic object. Also, infants’ greater interest in others’ goals, which develops between 3- and 30-months of age (Frank et al., 2012; Dalrymple et al., 2019), may be linked to both more selective attention to objects touched by a hand and successful encoding of a final spatial relation between objects. Future research that manipulates the attention to objects (for example, using flickering lights) would clarify the causal relationship. Also, this study tested only 8-month-old infants, leaving it unclear how attention to objects is related to relational processing for older infants such as 14 months. Fourteen-month-olds failed to form an abstract category of support relations with object sets of increased variability (Casasola, 2005b). Possibly, attention to relevant objects is less needed for older infants’ categorization of spatial relations, as their skills to selectively attend to spatial relations are more developed (Ridderinkhof and van der Stelt, 2000). Furthermore, older infants’ greater interest in objects themselves may pose challenges in categorization of spatial relations by hindering their familiarization to the objects. Further investigation is needed for a deeper understanding of the influence of attention to objects on infant spatial learning. In the present study, we made predictions about measures in which the categorizers and non-categorizers would differ. However, in the huge data we did not analyze, there might be differences in visual attention patterns between the two groups that we could not predict. Recently, analyzing eye movement data with machine learning techniques has been attempted to discover the age-related differences in infants’ looking behaviors (Dalrymple et al., 2019) and to classify learning profiles during adults’ problem-solving (Sáiz-Manzanares et al., 2021). Future studies may use machine learning to reveal unpredicted differences between categorizers and non-categorizers.

To conclude, this study is the first that collected data on infants’ eye movements during a spatial categorization task to our knowledge. It provides direct evidence that infants’ attention to objects relates to their categorization of spatial relations in a positive way. It also suggests that infants’ focused attention to an object in motion may be a key characteristic that influences young infants’ learning of spatial categories. These findings offer insights into the mechanisms of infant spatial categorization by questioning the idea that object information and spatial relational information compete for infants’ attention. Along with the previous eye-tracking studies on spatial reasoning (see Nazareth et al., 2016, for examples), this study reveals the strength of the eye movement analyses as a tool for exploring spatial learning mechanisms.

## Data availability statement

The raw data supporting the conclusions of this article will be made available by the authors, without undue reservation.

## Ethics statement

The studies involving humans were approved by the Seoul National University Institutional Review Board. The studies were conducted in accordance with the local legislation and institutional requirements. Written informed consent for participation in this study was provided by the participants’ legal guardians/next of kin.

## Author contributions

JC: Formal analysis, Visualization, Writing−original draft, Writing−review and editing, Investigation, Project administration. YP: Conceptualization, Funding acquisition, Resources, Writing−original draft, Writing−review and editing, Investigation, Supervision.
