# Supplementary material for: What is moving where? Infants’ visual attention to dynamic objects may assist with processing of spatial relations
Source: Front Psychol. 2024 Jan 18;14:1261201. doi: 10.3389/fpsyg.2023.1261201 (PMC10830844; doi:10.3389/fpsyg.2023.1261201)
Supplement: Supplementary file 1 [file Presentation_1.pdf]

What is Moving Where? Infants' Visual Attention to Dynamic Objects May Assist with Processing of Spatial Relations

Supplementary Material

Jihye Choi[1] and Youjeong Park[1*]

[1]Department of Child Development and Family Studies, Seoul National University, Seoul, South Korea

[*]Correspondence: Youjeong Park

youjeongpark@snu.ac.kr

Here we present the results from fixation analyses on the three test trials: the familiar object familiar relation (FoFr) trial, the familiar object novel relation (FoNr) trial, and the novel object familiar relation (NoFr) trial.

We examined whether the categorizers and non-categorizers differed in their *number* of fixations on the dynamic and the stationary objects during the three test trials. In the FoFr trial, categorizers and non-categorizers did not differ in their proportionate number of fixations on the dynamic object ($M_{cat} = .40$, $SD_{cat} = .22$, $M_{non\text{-}cat} = .36$, $SD_{non\text{-}cat} = .25$, $t < 1$). The proportionate number of fixations on the stationary object also showed no group difference in this test trial ($M_{cat} = .04$, $SD_{cat} = .08$, $M_{non\text{-}cat} = .03$, $SD_{non\text{-}cat} = .06$, $t < 1$). In the FoNr trial, categorizers' and non-categorizers' proportionate numbers of fixations on the dynamic object did not differ ($M_{cat} = .36$, $SD_{cat} = .28$, $M_{non\text{-}cat} = .26$, $SD_{non\text{-}cat} = .18$, $t(26) = 1.27$, p = n.s.). Their proportionate number of fixations on the stationary object did not differ, either ($M_{cat} = .15$, $SD_{cat} = .21$, $M_{non\text{-}cat} = .14$, $SD_{non\text{-}cat} = .15$, $t < 1$, p = n.s.). In the NoFr trial, categorizers' and non-categorizers' proportionate numbers of fixations on the dynamic object were approximately equal ($M_{cat} = .45$, $SD_{cat} = .23$, $M_{non\text{-}cat} = .44$, $SD_{non\text{-}cat} = .24$, $t < 1$). So were their proportionate numbers of fixations on the stationary object ($M_{cat} = .03$, $SD_{cat} = .06$, $M_{non\text{-}cat} = .03$, $SD_{non\text{-}cat} = .05$, $t < 1$).

We also analyzed infants' proportionate *duration* of fixations. Similar to the findings from the analyses of proportionate numbers of fixations, no significant difference was found between categorizers and non-categorizers. More specifically, in FoFr, categorizers and non-categorizers did not differ in their proportionate duration of fixations on the dynamic object ($M_{cat} = .42$, $SD_{cat} = .25$, $M_{non\text{-}cat} = .35$, $SD_{non\text{-}cat} = .25$, $t < 1$). The two groups' proportionate durations of fixations on the stationary object were also approximately equal in this test trial ($M_{cat} = .02$, $SD_{cat} = .04$, $M_{non\text{-}cat} = .03$, $SD_{non\text{-}cat} = .05$, $t < 1$). In FoNr, categorizers' and non-categorizers' proportionate durations of fixations on the dynamic object did not significantly differ ($M_{cat} = .32$, $SD_{cat} = .27$, $M_{non\text{-}cat} = .23$, $SD_{non\text{-}cat} = .20$, $t(29) = 1.07$, p = n.s.); neither did their proportionate durations of fixations on the stationary object ($M_{cat} = .15$, $SD_{cat} = .21$, $M_{non\text{-}cat} = .09$, $SD_{non\text{-}cat} = .10$, $t(23) = 1.02$, p = n.s.). In NoFr, again, categorizers' and non-categorizers' proportionate durations of fixations on the dynamic object were approximately equal ($M_{cat} = .45$, $SD_{cat} = .26$, $M_{non\text{-}cat} = .44$, $SD_{non\text{-}cat} = .25$, $t < 1$), and

so were their proportionate durations of fixations on the stationary object (M_{cat} = .02, SD_{cat} = .04, $M_{non\text{-}cat}$ = .03, $SD_{non\text{-}cat}$ = .06, $t < 1$). Note that the sample size differed by trial (FoFr: n_{cat} = 17, $n_{non\text{-}cat}$ = 20; FoNr: n_{cat} = 17, $n_{non\text{-}cat}$ = 19; NoFr: n_{cat} = 15, $n_{non\text{-}cat}$ = 19) due to the exclusion of infants whose fixation data were not recorded in each trial, resulting in fluctuations in degrees of freedom.

We then examined the relative distribution of attention between the dynamic and stationary objects in categorizers and non-categorizers. Categorizers consistently fixated longer on the dynamic object than the static object in all three test trials (FoFr: paired t (16) = 6.49, p < .001, FoNr: t (16) = 2.12, p = .050, NoFr: t (14) = 6.38, p < .001; see Supplementary Figure 1). The asymmetric distribution of attention was also found in categorizers' proportionate number of fixations: in all test trials, they allocated more fixations on the dynamic than the static object (FoFr: t (16) = 5.72, p < .001, FoNr: t (16) = 2.76, p = .01, NoFr: t (14) = 7.23, p < .001). In contrast, our non-categorizer infants displayed inconsistent patterns in relative allocation of fixations to the objects. They fixated longer (FoFr: paired t (19) = 5.52, p < .001, NoFr: t (18) = 6.82, p < .001) and allocated more fixations on the dynamic than the static object (FoFr: t (19) = 5.65, p < .001, NoFr: t (18) = 7.20, p < .001) in the test trials with familiar relations. However, in the test trial with a novel relation (FoNr), their fixations on the dynamic versus static objects differed only in the proportionate duration, t (18) = 2.43, p = .03, but not in the proportionate number, t (18) = 1.98, p = n.s, providing weaker evidence of attentional focus favoring the dynamic object over the stationary one during the novel relation trial.

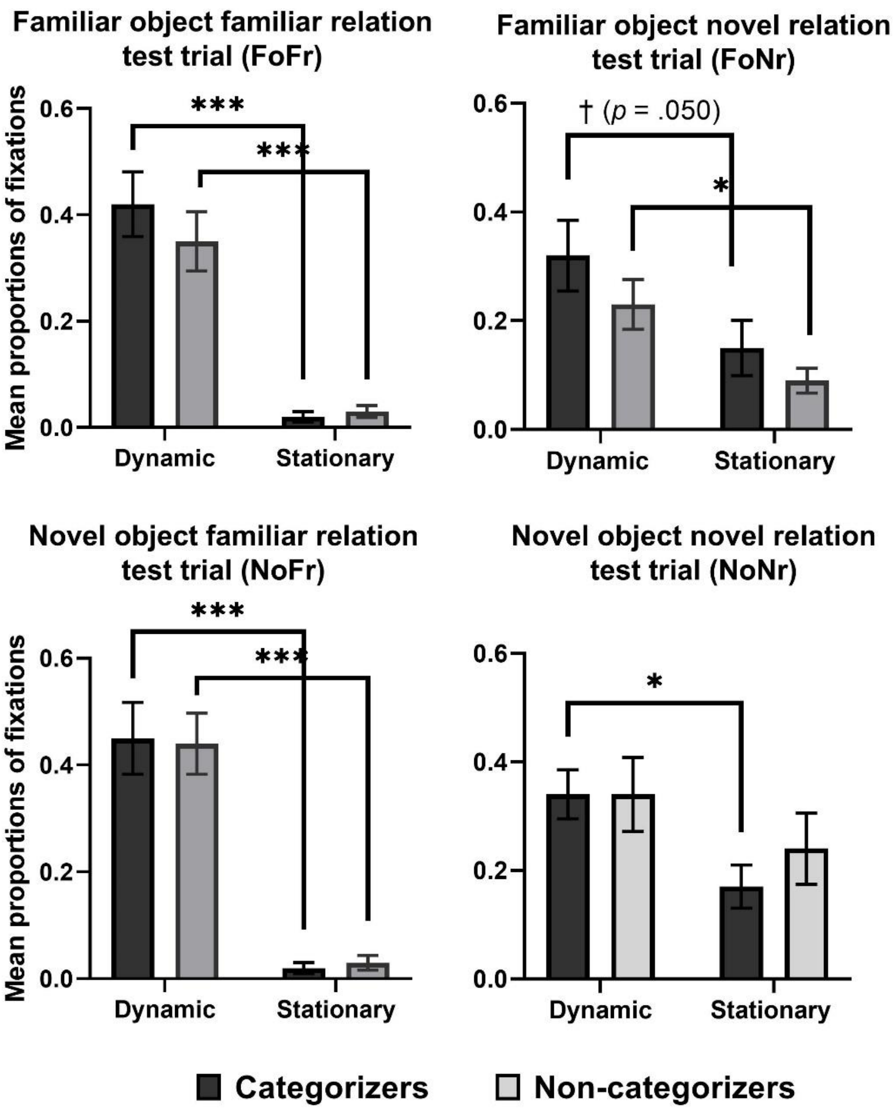

Supplementary Figure 1. The mean proportions of fixation duration on the dynamic and stationary objects during the test trials by group. Error bars indicate ±1 standard error of the means. † $p < .10$, * $p < .05$, *** $p < .001$. The analyses on the proportionate number of fixations yielded similar results regarding statistical significance, except for the FoNr trial. During the FoNr trial, only categorizers showed a significant difference in proportionate number of fixations on the dynamic versus stationary objects.
